# Resveratrol and Its Derivatives Diminish Lipid Accumulation in Adipocytes In Vitro—Mechanism of Action and Structure–Activity Relationship

**DOI:** 10.3390/nu16223869

**Published:** 2024-11-13

**Authors:** Noémi Sikur, Csenge Böröczky, Alexandra Paszternák, Ramá Gyöngyössy, Éva Szökő, Kamilla Varga, Tamás Tábi

**Affiliations:** 1 Department of Pharmacodynamics, Semmelweis University, 4 Nagyvárad Tér, H-1089 Budapest, Hungarypaszternak.alexandra@semmelweis.hu (A.P.); szoko.eva@semmelweis.hu (É.S.); varga.kamilla@phd.semmelweis.hu (K.V.); 2 Center for Pharmacology and Drug Research & Development, Semmelweis University, 26 Üllői Út, H-1085 Budapest, Hungary

**Keywords:** obesity, adipocyte, resveratrol, lipid accumulation, caloric restriction

## Abstract

Background and Objectives: Expansion of white adipose tissue causes systemic inflammation and increased risk of metabolic diseases due to its endocrine function. Resveratrol was suggested to be able to prevent obesity-related disorders by mimicking caloric restriction; however, its structure–activity relationships and molecular targets are still unknown. We aimed to compare the effects of resveratrol and its analogues on adipocyte metabolism and lipid accumulation in vitro. Methods: Mouse embryonic fibroblasts were differentiated to adipocytes in the absence or presence of resveratrol or its derivatives (oxyresveratrol, monomethylated resveratrol, or trimethylated resveratrol). Intracellular lipid content was assessed by Oil Red O staining. Glucose uptake and its response to insulin were estimated by 2-NBDG, and mitochondrial activity was assayed via resazurin reduction. Involvement of potential molecular pathways was investigated by concurrent treatment with their inhibitors. Results: Although lipid accumulation was significantly reduced by all analogues without altering protein content, oxyresveratrol was the most potent (IC50 = 4.2 μM), while the lowest potency was observed with trimethylated resveratrol (IC50 = 27.4 μM). Increased insulin-stimulated glucose uptake was restored by each analogue with comparable efficiency. The enhanced mitochondrial activity was normalized by resveratrol and its methylated derivatives, while oxyresveratrol had a minor impact on it. Among the examined pathways, inhibition of SIRT1, PGC-1α, and JNK diminished the lipid-reducing effect of the compounds. Autophagy appeared to play a key role in the effect of all compounds but oxyresveratrol. Conclusions: Resveratrol and its analogues can mimic caloric restriction with complex mechanisms, including activation of SIRT1, PGC-1α, and JNK, making them possible drug candidates to treat obesity-related diseases.

## 1. Introduction

In the last few decades, obesity has been referred to as a modern epidemic, with its prevalence constantly increasing at a fast pace [1]. According to the World Health Organization, in 2022, 43% of adults aged 18 years and above were overweight, and 16% were living with obesity [2]. The disease is characterized by the altered expansion and remodeling of body fat [3], with white adipose tissue containing dysfunctional adipocytes and an enhanced proportion of infiltrated immune cells [4,5]. These alterations in cellular composition lead to the secretion of cytokines, including TNFα and IL-6 [5,6]. Moreover, the depletion of lipid storage capacity causes increased lipotoxicity, affecting other metabolic functions [4,7]. Due to the development of low-level chronic inflammation, obesity can be accompanied by metabolic and cardiovascular diseases, such as insulin resistance, type 2 diabetes, hypertension, or atherosclerosis [8]. The disrupted metabolic homeostasis results in impaired quality of life and decreased life expectancy and requires complex treatment with high healthcare expenditure, sometimes in addition to surgical intervention [9,10].

The outcome of obesity-related health problems is impaired as the mass of white adipose tissue increases in response to environmental or genetic signals [11]. Generally, energy surplus is known to induce adipocyte hyperplasia and hypertrophy, both tightly regulated processes to protect other tissues from damage caused by high levels of glucose and triglycerides. Peroxisome proliferator-activated receptor gamma (PPARγ) and CCAAT/enhancer-binding proteins (C/EBP) are the main regulators of the expression of adipocyte-specific genes. Fat cells, however, are also highly dependent on insulin signaling, mainly through the PI3K/Akt pathway, to transform and mature [12,13]. Although white adipose tissue exhibits remarkable plasticity, enlarged adipocytes show a gradual loss of function. Extensive hypertrophy causes defection in GLUT4 translocation and, therefore, in insulin-stimulated glucose uptake [4,12]. Various proteins with pleiotropic functions are also involved in both normal and pathological regulation of adipocyte differentiation and proliferation, as well as adipose tissue inflammation, including ERK1, p38, and JNK1. These mitogen-activated protein kinases (MAPKs) stimulate adipogenesis through the regulation of gene transcription. The activity of ERK is mainly associated with the early stages of adipogenesis, as it inhibits PPARγ expression during proliferation, while p38 is rather involved in the browning of white adipose tissue along with UCP1 and PGC-1α proteins. The TNFα-modulating effect of MAPKs was reported, resulting in the expression of proinflammatory proteins and metabolic inflammation [14,15,16]. Mice lacking JNK1 and ERK1 were shown to be resistant to excessive adiposity and insulin resistance, which indicates the major role of these proteins in the development of obesity-related diseases [17,18]. Increased activity of p38 was observed in type 2 diabetic adipocytes, which might be linked to low levels of GLUT4 in cells [17].

Given the evidence that, in humans, dietary habits appear to be a crucial factor in delaying the onset of age-related diseases and achieving a prolonged health span, there has been considerable interest in the potential benefits of caloric restriction [19]. This has been prompted by the observations of lifespan extension in underfed laboratory rodents with a concomitant reduction in the incidence of age-related disorders [20]. The molecular basis of caloric restriction is an intensively researched topic that has yielded many results in recent years. It was found that this mild biological stress regulates adipogenesis and lipid accumulation through energy-sensing molecules, including AMPK, SIRT1, PGC-1α, mTOR, and insulin signaling pathways. This causes modulation of PPARγ activity and enhances mitochondrial biogenesis, β-oxidation, thermogenesis, and autophagy. These molecular mechanisms prevent excessive adipose tissue proliferation and repair its disrupted metabolic functions. Therefore, they have been widely studied as possible therapeutic targets [21,22,23]. Several dietary polyphenols have sparked great attention in aging research due to apoptosis-modulating, autophagy-inducing, antioxidant, and mitochondria-protecting effects that might indicate their positive impact on longevity [24].

Resveratrol (trans-3,4′,5-trihydroxystilbene), a naturally occurring non-flavonoid polyphenol classified in the group of stilbenes, is found in sources such as grapes, peanuts, berries, and red wine. In plants, it is synthesized as a response to biotic stress like fungal infection or abiotic stress, including UV radiation [25]. Resveratrol became well-known when the French paradox was first associated with this phytochemical [26], and since then, a wide range of studies have demonstrated its antioxidant, anti-inflammatory, and cardioprotective effects [27]. Recently, it has been suggested to have a potent antiadipogenic and metabolic-homeostasis-repairing effect [27,28,29] by mimicking caloric restriction in a SIRT1/AMPK-related pathway [30]. Its mechanism of action, molecular targets, and structure–activity relationships, however, are only partially understood. Despite the availability of resveratrol as a dietary supplement, in in vivo models, its bioavailability is relatively low, as it is rapidly metabolized by conjugation on its three hydroxyl groups in enterocytes and the liver [31]. Clinical studies have also been conducted to analyze the pharmacokinetic properties of resveratrol in humans. Although the 500 mg oral dose was well-absorbed, low plasma concentrations were detected in volunteers with higher levels of glucuronated and sulfated resveratrol [32]. Improved metabolic stability and bioavailability of resveratrol analogues with methylated hydroxyl groups was previously shown [33,34]. The hydroxyl substituents, however, may significantly contribute to the pharmacodynamic properties of stilbenes as well, and their involvement in the antiobesity effect of resveratrol is yet unclear. Therefore, extensively examining structurally modified analogues might result in finding a potential drug candidate with better efficiency and bioavailability.

The present study was conducted to compare the metabolic effects of resveratrol and its three structurally modified analogues with different numbers of free hydroxyl groups, oxyresveratrol (trans-3,4′,5,2′-tetrahydroxystilbene) and monomethylated (trans-3-methoxy-4′,5-dihydroxystilbene) and trimethylated resveratrol (trans-3,4′,5-trimethoxystilbene), on adipocyte differentiation and lipid accumulation. We also aimed to better understand the main molecular mechanism underlying the inhibition of adipogenesis and to identify the potential targets in the case of each individual compound. The main focus was on insulin signaling and mitochondria-related pathways, which are also activated by caloric restriction. We intended to study structure–activity relationships by including hydroxylated and methylated analogues in the experiments.

## 2. Materials and Methods

### 2.1. Materials

Resveratrol, oxyresveratrol, and monomethylated and trimethylated resveratrol were obtained from Tokyo Chemical Industry (TCI, Tokyo, Japan). Fetal bovine serum (FBS) was purchased from BioSera (Nuaille, France), and DMEM, trypsin, and stable glutamine were supplied by Corning Inc. (New York, NY, USA). Dexamethasone, 3-isobuthyl-1-methylxanthine, resazurin, 2-deoxy-2-(7-nitro-2,1,3-benzoxadiazol-4-yl)amino-D-glucose (2-NBDG), Hoechst 33342, Brilliant Blue R-250, and Oil Red O dye were obtained from ThermoFisher Scientific (Waltham, MA, USA). Insulin was provided by University Pharmacy of Semmelweis University (Budapest, Hungary). Rosiglitazone was obtained from BLD Pharmatech GnbH (Kaiserslautern, Germany). Wortmannin, SB202190, EX-527, SR18292, chloroquine, PD98059, and SR500125 were purchased from MedChemExpress (Monmouth Junction, NJ, USA).

Test compounds and other reagents were dissolved in dimethyl sulfoxide (DMSO) and the final concentration in cell cultures did not exceed 0.5% in any of the experiments.

To establish cell cultures, pregnant NMRI mice were supplied by Toxicoop (Gödöllő, Hungary). We adhered to the EU Directive 86/609/EEC regarding the use of laboratory animals in scientific research.

### 2.2. Cell Differentiation

Mouse embryonic fibroblast (MEF) cell culture was established according to a CSH protocol [35]. The cells were seeded at a density of 40,000 cells/cm^2^ and cultured in DMEM containing 10% fetal bovine serum, 1% non-essential amino acids, 1% stable glutamine, 80 µg/mL gentamicin, and 10 µg/mL ciprofloxacin and were used between passages 3 and 6. Two days after reaching confluency, differentiation was induced by a medium containing 100 ng/mL insulin, 1 µM rosiglitazone, 0.2 mM dexamethasone, and 0.5 µM 3-isobuthyl-1-methylxanthine in all groups, including the control one. Adipogenesis was maintained with 100 ng/mL insulin and 1 μM rosiglitazone for another 17 days, except the control group. During the maintenance phase, cells were treated with resveratrol analogues (resveratrol, oxyresveratrol, monomethylated or trimethylated resveratrol) in various concentrations or vehicle, in the presence or absence of molecular pathway inhibitors. Culture medium was replaced every second day. The effect of resveratrol derivative treatment was compared to control cells subjected to only 4-day induction, followed by culturing in DMEM/FBS medium, as well as to a differentiated group that underwent the complete differentiation protocol.

### 2.3. Oil Red O and Coomassie Blue Staining for Detection of Lipid and Protein Content

Cells were differentiated in 24-well plates, with simultaneous treatment with 0, 1.56, 3.125, 6.25, 12.5, 25, 50, or 100 μM resveratrol, oxyresveratrol, monomethylated or trimethylated resveratrol, respectively. Following fixation with 4% paraformaldehyde, the cells were stained with saturated Oil Red O solution (in 60% isopropanol) for 20 min. Lipid accumulation was visually observed by using a phase-contrast microscope. In order to quantify the result, the stain, retained in lipid droplets, was extracted into isopropanol, and the absorbance of the resulting solutions was measured at a 518 nm wavelength by using a Varioskan LUX microplate reader (ThermoFisher Scientific).

After removal of Oil Red O stain, protein content of cultures was quantified to obtain information about incidental cytotoxic effect. The cells were stained by a 0.1% solution of Coomassie Blue in 10% acetic acid, 50% methanol solution for 10 min. The stain, bound to proteins, was redissolved into methanol, and the absorbance of the resulting solutions was measured at a 595 nm wavelength by a Varioskan LUX microplate reader.

### 2.4. Measurement of Mitochondrial Activity

The effect of resveratrol and its analogues on mitochondrial activity was assessed via resazurin reduction in the mitochondria by dehydrogenase enzymes. Cells were differentiated in 96-well plates and treated with 4 different concentrations: 3.125, 6.25, 12.5, and 50 μM of resveratrol analogues. After administration of 0.015 mg/mL resazurin reagent, the cells were incubated for 4 h, and the fluorescence intensity was measured at 530/590 nm by using a Varioskan LUX microplate reader.

### 2.5. Assessment of Glucose Uptake

Glucose uptake of the cells was measured by 2-deoxy-2-(7-nitro-2,1,3-benzoxadiazol-4-yl)amino-D-glucose (2-NBDG), a fluorescent glucose analogue, in 96-well plates after treatment with 25 μM of resveratrol derivatives. Changes in basal and insulin-stimulated glucose uptake were assessed. After incubating the cells with 0, 10, 100, 1000 pM insulin for 1 h, HBSS buffer containing 200 μM 2-NBDG was added into each well for another hour. Simultaneously, 2 nM Hoechst 33342 DNA staining was used to normalize the rate of glucose uptake to cell number. Fluorescence of 2-NBDG and Hoechst 33342 was recorded at 465/538 nm and 350/461 nm, respectively, by a Varioskan LUX microplate reader.

### 2.6. Investigating Molecular Pathways of Resveratrol

To clarify the molecular mechanisms in the effect of resveratrol analogues, some groups were simultaneously treated with specific inhibitors of potential molecular targets (PI3K: wortmannin 2.5 μM, SIRT1: EX-527 2 μM, p38: SB202190 10 μM, PGC-1α: SR18292 10 μM, autophagy: chloroquin 0.1 μM, ERK: PD98059 5 μM, or JNK: SP600125 5 μM) during the maintenance phase. The effect of resveratrol analogues in the presence of inhibitors on lipid accumulation was investigated after 21 days’ incubation by the Oil Red O/Coomassie Blue staining and spectrophotometric measurement, as described in Section 2.3.

### 2.7. Statistical Analysis

Our results were evaluated by using GraphPad Prism 8.0 data analysis software (GraphPad Software, La Jola, CA, USA). Data are expressed as mean ± SD of 3–4 parallel measurements. Concentration–response curves were plotted using non-linear regression with the least squares method. Data were analyzed for normality by the Shapiro–Wilk test and for homoscedasticity by the Brown–Forsythe test. All data were involved in the statistical analysis, as no outliers were identified by the ROUT method. For the study of mitochondrial activity and glucose uptake, data were evaluated by one-way ANOVA followed by Dunnett’s post hoc test to compare each group to the differentiated group. During molecular target assay, results were analyzed by two-way ANOVA and Tukey’s post hoc test to compare every group with the others.

## 3. Results

### 3.1. Effects of Resveratrol Analogues on Lipid Accumulation and Protein Levels of Adipocytes

**Figure 1:**
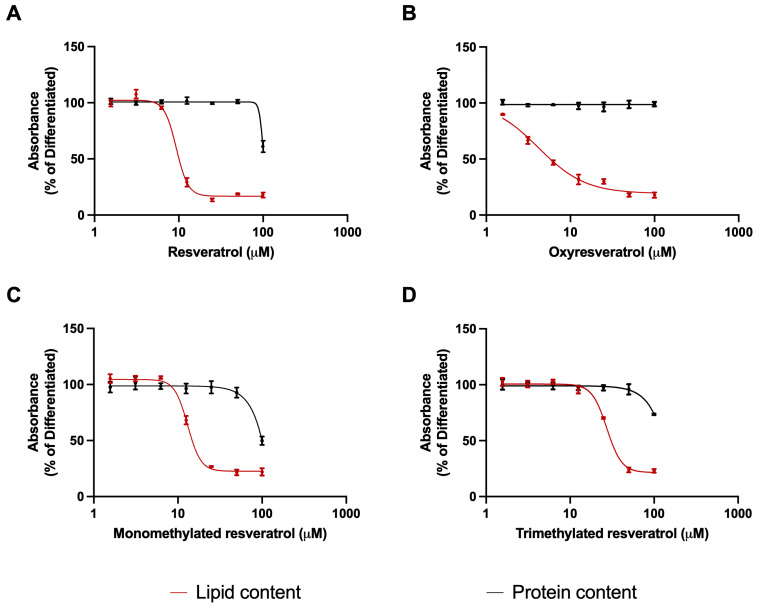
Effect of resveratrol derivatives on lipid accumulation and protein levels. Treatments were carried out in 1.56, 3.125, 6.25, 12.5, 25, 50, and 100 μM concentrations in differentiation-maintaining medium for 17 days after a 4-day initiation period. The lipid and protein contents were measured by Oil Red O and Coomassie Blue staining, respectively. According to spectrophotometric measurements, absorbance of control group was 0.0607 ± 0.00329, while differentiated group showed 0.2067 ± 0.005735 absorbance units. Oxyresveratrol (**B**) decreased lipid accumulation with the highest potency (IC50 = 4.15 μM, 95% CI: 2.80–5.16), followed by resveratrol (**A**) (IC50 = 9.37 μM, 95% CI: 8.89–9.91) and monomethylated resveratrol (**C**) (IC50 = 13.38 μM, 95% CI: 12.74–14.04). Trimethylated derivative (**D**) reduced lipid levels with the lowest potency (IC50 = 27.39 μM, 95% CI: 26.41–28.48). All data are expressed in the percentage of lipid and protein content of the untreated differentiated group, non-linear regression with least squares method was used for plotting.

The characteristics of adipocytes were observed under a phase-contrast microscope, including a spherical shape and the appearance of red intracellular lipid droplets after Oil Red O staining in response to differentiation. In contrast, cultures treated with resveratrol derivatives showed a reduction in the amount and size of red spots, accompanied by a decrease in the number of fibroblast cells transforming into adipocytes. Based on spectrophotometric measurement of retained Oil Red O stain, each analogue induced a concentration-dependent reduction in the lipid accumulation with similar efficacy but different potency. Oxyresveratrol was the most potent in reducing lipid levels, with a more elongated concentration–response curve. Resveratrol and monomethylated resveratrol inhibited adipogenesis with intermediate potency, and the trimethylated derivative was the least potent one, indicated by its higher IC50 value. Protein levels were reduced only at the highest concentration of 100 μM, with the exception of oxyresveratrol, where the potent effect on lipid accumulation was not accompanied by a decrease in protein content at all (Figure 1).

### 3.2. Effects of Resveratrol Analogues on Mitochondrial Activity

**Figure 2:**
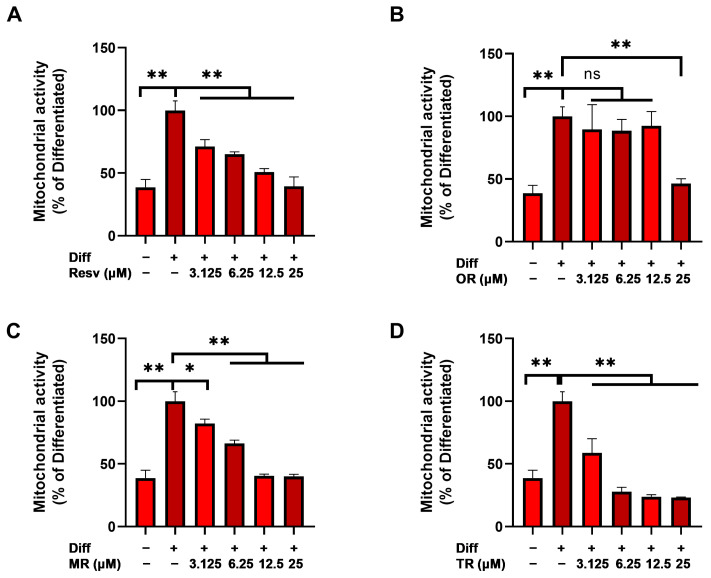
Effect of resveratrol derivatives on elevated mitochondrial activity measured by resazurin reduction assay. After a 4-day initiation period, cells were treated with 3.125, 6.25, 12.5, and 25 μM of resveratrol derivatives for 17 days during maintenance. Compared to the differentiated group, (**D**) trimethylated derivative reduced the activity by 76.24% in 12.5 μM concentration. In the same concentration, treatment with (**A**) resveratrol and (**C**) monomethylated resveratrol caused 49.24% and 59.53% reduction, respectively. However, (**B**) 12.5 μM oxyresveratrol treatment decreased the activity only by 7.52%. Results were analyzed by one-way ANOVA with Dunnett’s post hoc test. Diff: differentiation, Resv: resveratrol, OR: oxyresveratrol, MR: monomethyl resveratrol, TR: trimethyl resveratrol, ** *p* < 0.0001, * *p* = 0.003, ns: not significant.

Resazurin, a redox-sensitive dye, was used to assess the rate of metabolic activity in mitochondria. Compared to the control group, the differentiated cells showed enhanced activity, which was decreased by all the derivatives. However, the extent of the effect varied depending on the structure of the compounds. The potency of trimethylated resveratrol was found to exceed that of the others. Even at a 6.25 μM concentration, it restored the activity to the control level, while oxyresveratrol showed no effect on mitochondrial activity apart from the highest studied concentration (25 μM) (Figure 2).

### 3.3. Effects of Resveratrol Analogues on Glucose Uptake in the Presence and Absence of Insulin

**Figure 3:**
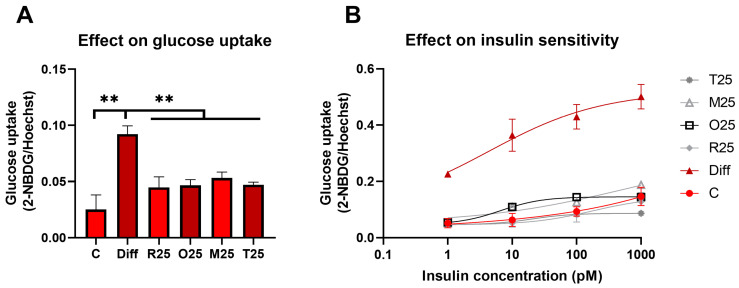
Effect of resveratrol derivatives on (**A**) glucose uptake and (**B**) insulin sensitivity of differentiated MEF cells. The derivatives were added to the medium for 17 days, then glucose uptake was estimated by 2-NBDG with or without prior incubation with 1, 10, 100, 1000 pM insulin for 1 h. Treatment with 25 μM resveratrol, oxyresveratrol, monomethylated and trimethylated resveratrol reduced the increased glucose uptake by 51.4%, 49.4%, 42.3%, and 48.8%, respectively (**A**). The enhanced insulin sensitivity of adipocytes was abolished by each derivative with similar efficiency (**B**). The obtained data were evaluated by (**A**) one-way ANOVA with Dunnett’s post hoc test and (**B**) non-linear regression with least squares method. ** *p* < 0.0001.

In line with our results on mitochondrial activity, the basal glucose uptake significantly increased after differentiation. All resveratrol derivatives, at a 25 μM concentration, normalized the glucose uptake with similar efficiency. Following the induction of adipogenesis, an enhanced insulin sensitivity was observed, which was restored by resveratrol and all three analogues, with comparable efficiency (Figure 3).

### 3.4. Effects of Resveratrol Analogues on Lipid Accumulation and Protein Content in the Presence of Inhibitors of Molecular Pathways

Specific inhibitors were used to investigate the involvement of the potential molecular targets in the mechanism of action. Intracellular lipid and protein levels were compared by Oil Red O and Coomassie Blue staining, respectively, followed by spectrophotometric measurement.

**Figure 4:**
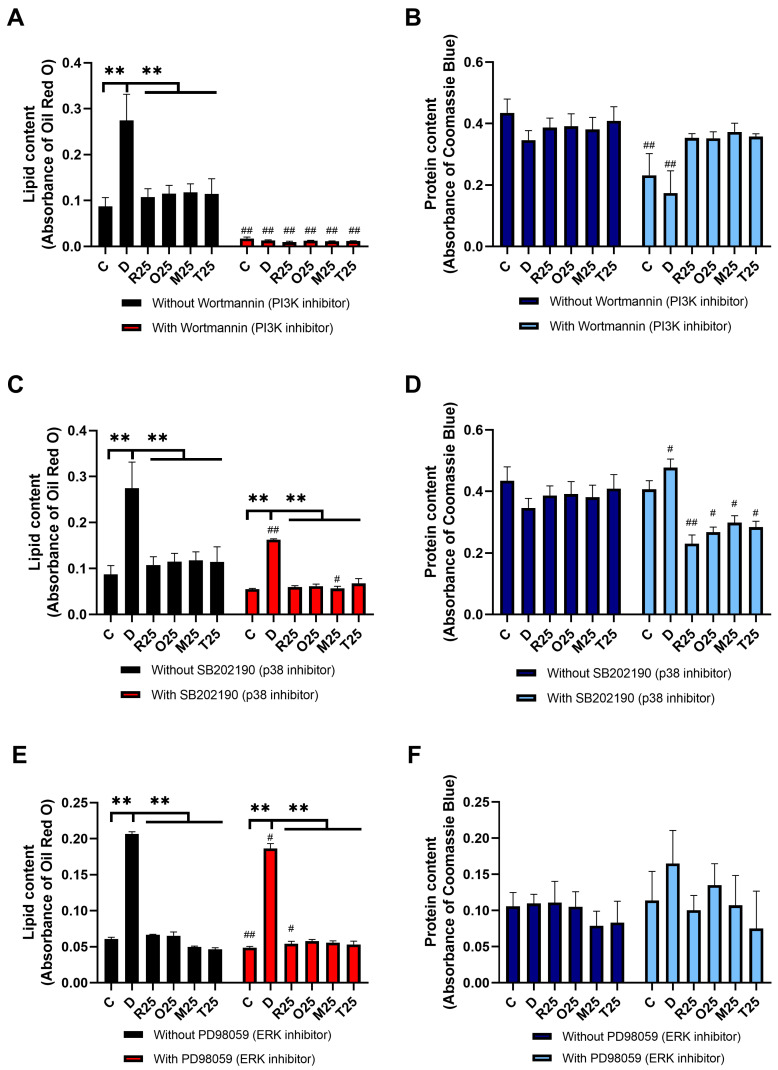

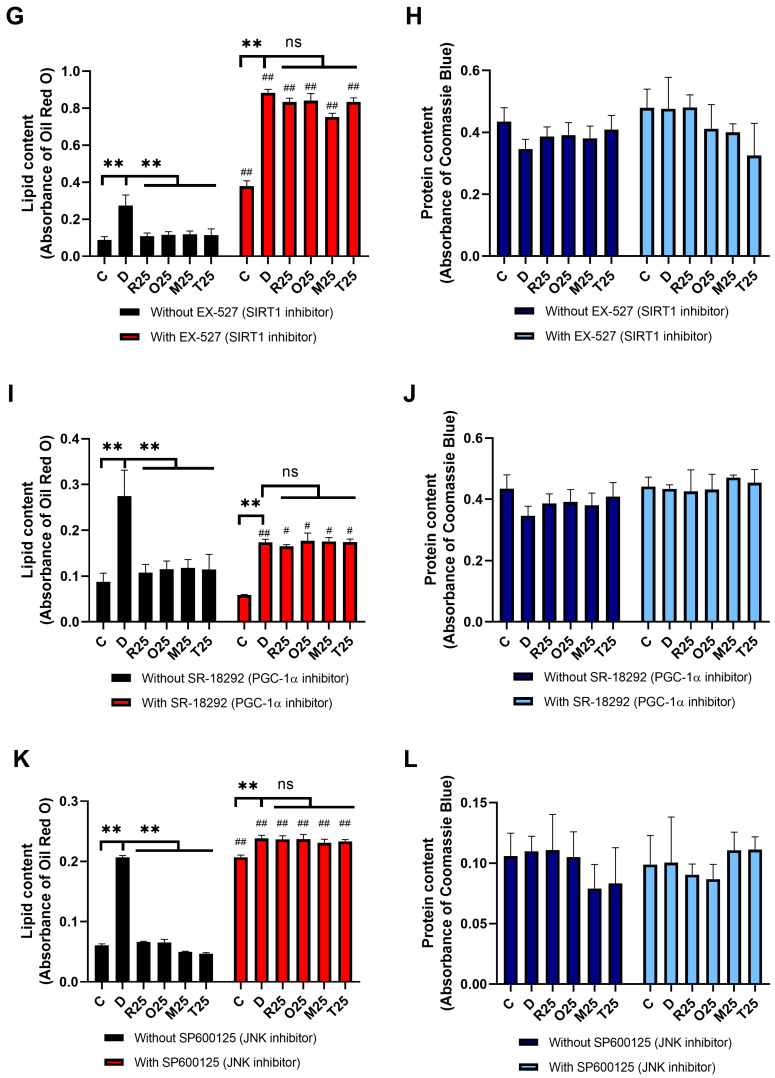

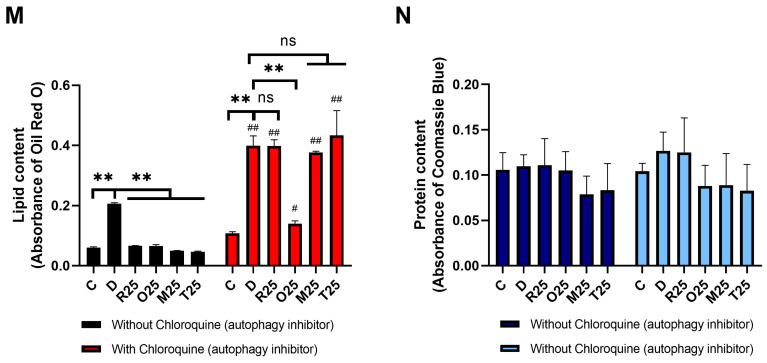
The effect of resveratrol derivatives on lipid accumulation and protein levels in the absence and presence of PI3K (**A**,**B**), p38 (**C**,**D**), ERK (**E**,**F**), SIRT1 (**G**,**H**), PGC-1α (**I**,**J**), JNK (**K**,**L**), or autophagy (**M**,**N**) inhibition. Resveratrol derivatives were used in 25 μM concentration in differentiating medium for 17 days and the cells were simultaneously treated with inhibitors of the potential targets: 2.5 μM wortmannin, 2 μM EX-527, 10 μM SR-18292, 10 μM SB202190, 0.1 μM chloroquine, 5 μM SP600125, or 5 μM PD98059. The lipid and protein levels were assessed by Oil Red O and Coomassie Blue staining, respectively. Inhibition of PI3K (**A**) obliterated adipogenesis in the differentiated groups. SIRT1 (**G**), autophagy (**M**), and JNK (**K**) inhibition significantly increased the lipid levels, while blocking PGC-1α (**I**), p38 (**C**), and ERK (**E**) activity resulted in reduced lipid droplet formation. The lipid-reducing effect of resveratrol and its analogues disappeared with the inhibition of SIRT1 (**G**), PGC-1α (**I**), and JNK (**K**). The effect of the derivatives appeared to be dependent on autophagy, except for oxyresveratrol (**M**). Protein content was consistently reduced only by p38 inhibitor SB202190 (**D**). Results were evaluated by two-way ANOVA and Tukey’s post hoc test. ** *p* < 0.0001 vs. differentiated group, ^#^ *p* < 0.05, ^##^ *p* < 0.0001 vs. respective group without inhibitor treatment, ns: not significant.

When wortmannin, an inhibitor of PI3K, was used, lipid accumulation was obliterated in both the differentiated and the treated groups, therefore, the effect of resveratrol analogues on adipogenesis could not be investigated (Figure 4A,B).

Inhibition of p38 kinase by SB202190 resulted in a decreased cellular lipid content in all groups. However, the inhibitory effect of the analogues on the lipid accumulation remained significant (Figure 4C). Furthermore, the protein levels were also reduced in response to SB202190 treatment (Figure 4D).

The repression of ERK activity by PD98059 did not alter protein contents, and the resveratrol derivatives still significantly reduced lipid accumulation compared to the differentiated group (Figure 4E,F).

Inhibition of SIRT1 by EX-527 (Figure 4G,H), PGC-1α by SR-18292 (Figure 4I,J), and JNK by SP600125 (Figure 4K,L) abolished the previously demonstrated inhibitory effect of resveratrol analogues on adipogenesis, as no significant difference in lipid accumulation was observed between the derivative-treated and the differentiated groups. Inhibition of these regulatory proteins in the absence of resveratrol derivatives differently affected the lipid levels of the matured adipocytes. In the absence of SIRT1 and JNK activity, lipid accumulation was markedly increased, whereas the inhibition of PGC-1α and ERK reduced lipid content in the differentiated cultures.

The addition of chloroquine, to block autophagy, significantly increased the lipid levels in the differentiated, as well as the resveratrol and monomethylated and trimethylated resveratrol, groups. On the other hand, oxyresveratrol significantly inhibited lipid accumulation even in the presence of the autophagy inhibitor (Figure 4M,N).

## 4. Discussion

The prevalence of obesity is continuously rising and is becoming a persistent global health concern [10]. The expansion of the white adipose tissue results in a phenotype switch, which is characterized by dysfunctional adipocytes, enhanced immune cell infiltration, and secretion of inflammatory cytokines. Its impaired function disrupts metabolic homeostasis, which links it to insulin resistance and type 2 diabetes [36]. The presence of comorbidities further complicates treatment, as polypharmacy is usually accompanied by several adverse effects [37]. New treatment options are being extensively researched and the current focus is mainly on prevention. Recent studies observed the caloric-restriction-mimetic effect of some naturally occurring compounds with antiadipogenic activity, including resveratrol [38], and the underlying molecular mechanisms have been widely investigated by in vitro and in vivo tests.

In this present study the adipocyte differentiation- and lipid accumulation-inhibiting effects of resveratrol derivatives on MEF cells were observed in vitro; however, the individual compounds showed different potency. SIRT1, PGC-1α, and JNK proteins were demonstrated to be key factors in their effects, while no significant role of p38 and ERK was found in the mechanism of action. Autophagy and mitochondria were identified as important targets of the derivatives, with the exception of the hydroxylated resveratrol analogue oxyresveratrol.

### 4.1. Involvement of Kinase Pathways in the Antiadipogenic Effect of Resveratrol Derivatives

In line with our results, Chen et al. also reported the lipogenesis-attenuating effect of resveratrol when used during differentiation of 3T3-L1 cells, which was accompanied by increased p-AMPKα expression. After treatment with AMPKα siRNA, an enhanced lipogenesis was observed, and the effect of resveratrol was completely abolished, which suggests the major role of AMPK in its mechanism of action [39]. In a similar study, a potent lipid-accumulation-abolishing effect of resveratrol during adipogenesis was observed by Rayalam et al. Both Chen et al. and Rayalam et al. also reported the apoptosis-inducing effect of higher concentrations of resveratrol on adipocytes, which may also contribute to its antiadipogenic effect [40]. Our Coomassie Blue staining results also confirm their data, as they suggest a decrease in cell number in response to 100 μM treatment by resveratrol or its analogues except for oxyresveratrol. The lower concentrations, however, did not affect protein levels, indicating the lack of significant cytotoxicity, as was found in our present experiments.

Because of its low bioavailability, resveratrol itself is not appropriate as a lead for drug research. Its metabolically more stable analogues containing methoxy instead of hydroxyl groups have thus attracted considerable attention. Chung and Hyun recently demonstrated the dose-dependent inhibitory effect of pinostilbene (monomethylated resveratrol) on lipid levels in 3T3-L1 cells, similarly to our present results [41]. According to their findings, pinostilbene normalized the increased phosphorylation of ERK, p38, and JNK, while in our study, the inhibition of ERK or p38 had no significant impact on the effect of resveratrol analogues. Therefore, we can speculate that the modulation they observed may rather be a consequence than a key step in the mechanism of action of the stilbenes. On the other hand, their and our results, regarding the involvement of JNK, are in good accordance. Phosphorylation of Akt during differentiation was also seen in the experiments of Chung and Hyun, while we demonstrated diminished lipid accumulation in response to the upstream PI3K inhibition in our present work, both indicating the major role of the PI3K/Akt pathway in adipogenesis.

### 4.2. Involvement of Insulin Signaling in the Antiadipogenic Effect of Resveratrol Derivatives

In our present study, resveratrol derivatives also reduced the enhanced glucose uptake and insulin sensitivity of the differentiated cells to the control level, with similar efficiency. The decreased glucose uptake and its reduced response to insulin are consistent with the inhibition of adipocyte formation, likely due to the prevention of their differentiation and maturation in response to resveratrol and resveratrol derivative treatment. A decrease in the number of matured adipocytes can reduce the expansion of adipose tissue and consequently the risk of obesity-related health issues. These findings are in line with the results reported by Lee and Kim on differentiated 3T3-L1 cells after short-term incubation with resveratrol in concentrations comparable to those used in our experiments [42]. On the other hand, Chen et al. observed an insulin-sensitivity-improving effect of resveratrol on dexamethasone-treated, insulin-resistant adipocytes after 24 h [43]. The discrepancy between their and our results may be explained by the difference in the cell culture model. In our experiment, the long-term inhibitory effect of resveratrol on adipogenesis was demonstrated, while the previous works examined its short-term effect on already differentiated adipocytes. Furthermore, contrary to the aforementioned study, we used rosiglitazone to establish a high insulin sensitivity characteristic that stimulated the development of an adipocyte phenotype.

### 4.3. Involvement of Mitochondria and Caloric Restriction Pathways in the Antiadipogenic Effect of Resveratrol Derivatives

Resveratrol might prevent obesity by enhancing thermogenesis by inducing the browning of the white adipose tissue. Li et al. recently reported that resveratrol induces a shift towards brown fat tissue phenotype in a SIRT1-dependent manner, which is accompanied by increased energy expenditure and heat production. Furthermore, the inferior efficacy of resveratrol metabolites, glucuronides and sulfate, was also shown, highlighting the importance of developing more metabolically stable analogues [44]. Indeed, a resveratrol derivative, oxyresveratrol, was reported by Choi et al., having a similar antiobesity effect associated with increased energy expenditure and enhanced expression of thermogenic genes, like UCP1, in white adipose tissue [45]. These results are in line with our observation regarding the reducing effect of resveratrol and its analogues on the mitochondrial activity. Furthermore, they have also found increased expression of PGC-1α, which is in accordance with our present study, where this protein was also proven to play a key role in the effect of resveratrol derivatives. The essential contribution of SIRT1 and PGC-1α to the lipid-content-reducing effect of resveratrol was also shown by Imamura et al., as the silencing of their genes was demonstrated to reverse the effect of resveratrol [46]. All these previous findings are in good accordance with our experiments using inhibitors, which indicate the crucial role of the SIRT1/PGC-1α pathway in the mechanism of action of resveratrol derivatives. SIRT1 and AMPK are known as metabolic sensors of cells, and both can be activated in response to energy scarcity. They are tightly connected to PGC-1α activity, which in turn affects mitochondrial biogenesis and the mobilization of lipid storage [47]. Considering our results and recent studies, resveratrol and its analogues might mimic caloric restriction on adipocytes through these pathways.

### 4.4. Involvement of Autophagy in the Antiadipogenic Effect of Resveratrol Derivatives

Resveratrol was reported to be an effective autophagy inducer by several research groups (for a recent review, see [48]). Previously, our group showed the essential role of autophagy induction in its cytoprotective effect on MEF cells [49,50]. Furthermore, our recent findings also revealed the structure-dependent activity of resveratrol derivatives; methylated analogues were effective autophagy inducers, while oxyresveratrol lacked this effect [51]. Morselli et al. suggested that resveratrol might also mimic caloric restriction by inducing autophagy through a SIRT1-dependent pathway [52], which is in good accordance with our present results of its abolished effect in the presence of SIRT1 or autophagy inhibitors. A sufficient rate of autophagy is required to maintain normal function of adipose tissue, as it has a complex role in adipocyte differentiation. Bulk autophagy is required in the process of adipogenesis [53], while a more specific form of it, lipophagy, contributes to mobilization of lipid droplets [54,55]. In accordance with the previous findings reporting a pivotal role of lipophagy in the reduction of lipid content of adipocytes, autophagy was found to be a key regulator of adipogenesis in our present study, as well. Its inhibition not only doubled lipid accumulation during adipocyte differentiation but also reversed the antiadipogenic effect of resveratrol and its methylated derivatives. Interestingly, the effect of oxyresveratrol on lipid accumulation was independent of autophagy according to our present results, which suggests the involvement of a distinct mechanism by which it can reduce adipogenesis. Lack of autophagy induction might be explained by its inferior effect on mitochondrial activity; however, better understanding of its mechanism of action requires further studies.

### 4.5. Limitations and Future Directions

This study has a few limiting factors. The measurements were performed on cell cultures isolated from mice; thus, the results should be translated with caution to the human clinical level. Treatment with molecular inhibitors of various pathways may trigger other compensatory effects, which might bias our findings on the mechanism of action. Further analysis is necessary to clarify the details of the complex mechanisms of action of the resveratrol analogues on adipocyte differentiation and adipogenesis. The contribution of modulation of inflammatory processes by resveratrol derivatives would add valuable pieces of information to our understanding.

## 5. Conclusions

Based on our results, we concluded that all the examined resveratrol derivatives diminished lipid accumulation of MEF cells with comparable efficiency. Nevertheless, considerable differences between their effects were also revealed. Despite its potent adipogenesis-inhibiting effect, mitochondria and autophagy appeared to be less relevant targets of the hydroxylated resveratrol derivative. Although trimethylated resveratrol inhibited lipid accumulation with less potency, it showed a stronger suppressing effect on the anabolic processes in the mitochondria. The results indicate that the alterations in the hydroxyl and methyl groups of the molecule may induce a shift in the dominant components of adipogenesis inhibition. Resveratrol and its analogues decreased glucose uptake and insulin sensitivity with similar efficiency, which further confirms their reducing effect on adipocyte formation. Pathways, activated by caloric restriction, are thought to be involved in the mechanism, but there may be differences in the molecular targets between individual analogues. A major role of SIRT1, PGC-1α, and JNK was identified in the lipid-accumulation-reducing effect. Our results indicate that in addition to resveratrol, its analogues, including the ones with better metabolic stability and oral bioavailability, can inhibit the maturation of adipocytes. Therefore, they can serve as lead molecules for further translational research. These observations could promote the therapeutic use of resveratrol derivatives in obesity prevention and treatment.

## Data Availability

The original contributions presented in the study are included in the article, further inquiries can be directed to the corresponding author.
